# Feasibility, safety, and short-term outcome of totally thoracoscopic mitral valve procedure

**DOI:** 10.1186/s13019-018-0819-1

**Published:** 2018-12-29

**Authors:** Qin Jiang, Tao Yu, Keli Huang, Lihua Liu, Xiaoshen Zhang, Shengshou Hu

**Affiliations:** 1 Department of Cardiac Surgery, Sichuan Provincial People’s Hospital, Affiliated Hospital of University of Electronic Science and Technology, No.32, West Second Section First Ring Road, Chengdu, China; 2 Department of Cardiac Surgery, Affiliated Hospital of University of Jinan, Guangzhou, China; 3 Department of Cardiac Surgery, Fuwai Hospital, Chinese Academy of Medical Sciences and Peking Union Medical College, Beijing, 100037 China

**Keywords:** Minimally invasive surgical procedures, Thoracoscopy, Mitral valve, Cardiac surgical procedures

## Abstract

**Background:**

The totally thoracoscopic procedure for mitral valve (MV) disease is a minimally invasive method. We investigated the procedure’s feasibility, safety and effectiveness when it was performed by an experienced operator.

**Methods:**

We retrospectively analysed 53 consecutive patients with MV disease treated between December 2014 and April 2017 by minimally invasive procedures. The procedures were performed on femoral artery-vein bypass through three 2–4 cm incisions, with one additional penetrating point on the right chest wall under totally thoracoscopic visual guidance and surveillance of transoesophageal echocardiography.

**Results:**

Two patients who underwent intraoperative conversion to sternotomy were excluded due to indivisible pleural cavity adhesion. Of the others (38 female patients, average age, 49 ± 14 years, left ventricular ejection fraction, 59 ± 7%), 34 received MV replacement for rheumatic mitral lesions, which was redone for one patient after the discovery of serious paravalvular leakage, 17 received MV repair for mitral regurgitation (with 4 secondary to atrial septum defect, 2 diagnosed with left atrial myxoma, and 2 redone for mitral valve replacement due to repair failure), 28 received additional tricuspid valvuloplasty, and one patient received a Warden procedure. The cardiopulmonary bypass and aortic cross clamp times were 144 ± 39 min and 80 ± 22 min, respectively. Postoperational chest tube drainage in the first 48 h was 346 ± 316 ml. The ventilation time and intensive care unit stay length were 11 ± 11 h and 23 ± 2 h, respectively. One patient died of disseminated intravascular coagulation and prosthesis thrombosis with fear of anticoagulation-related bleeding.

**Conclusions:**

The totally thoracoscopic procedure on mitral valves by an experienced surgeon is technically feasible, safe, effective and worthy of widespread adoption in clinical practice.

## Introduction

Accumulating knowledge of the structure, function, and pathology of the mitral valve (MV) has led to favourable surgical results in MV procedures. Advances in imaging and surgical instruments have allowed surgeons to perform less invasive sternum-sparing MV surgery [1]. Several kinds of minimally invasive access for MV procedures in adult patients have been popularly introduced into clinical practice. Lower hemisternotomy was used as a popular form of a minimally invasive MV procedure [2]. Right anterolateral minithoracotomy for MV surgery was a safe approach with very low operative mortality comparable to standard median sternotomy [3, 4]. MV surgery through a right minithoracotomy did not result in increased morbidity, mortality or procedural duration; therefore, long-term survival outcomes were satisfactory [5, 6].

Endeavours to reduce surgical trauma, hasten patient recovery, improve cosmetics, and increase patient satisfaction continued to further promote minimally invasive procedures. Additional minimally invasive surgical approaches, such as total video-assisted thoracoscopy or robotic assistance, have also been applied to repair congenital heart defects to minimize surgical trauma and improve cosmetic results [7–9]. Apart from comparable merits in cosmetics to robotic surgery, an approach using totally thoracoscopy assistance to repair MV without complicated hardware equipment has a remarkable advantage [10].

However, the application of totally video-assisted thoracoscopic procedures on MV has been very limited in clinical practice. It was demonstrated that adverse events, including intraoperative conversion to sternotomy, re-exploration for bleeding, and valve-related reoperation within the same hospital stay, occurred with a predicted 25% rate at the beginning; this trend remained sluggish with declines in conjunction with increased operation training [11]. In this study, we reported the two-year experience of our department regarding the totally endoscopic procedure on MV performed by a single experienced surgeon (Z.XS.).

## Materials and methods

### Patients

This was a single-centre, retrospective, observational study of prospectively collected data from consecutively recruited patients. This study was approved by the Institutional Review Board of Sichuan Provincial People’s Hospital. Written informed consent was preoperatively obtained from each participant and/or their parents or guardians, and the patients were fully informed about the technique and were able to choose a standard median sternotomy according to their preference. We initiated the protocol at the end of 2014, and since that time, totally endoscopic procedure on MV has become the preferred approach for selected patients with MV disease, no matter whether the disease was isolated or combined with tricuspid valve (TV) disease and atrial septal defect (ASD). The selection criteria were as follows: (1) no combination with serious aortic or coronary artery disease; (2) available to the operator; (3) no previous history of a right thoracotomy with expected pleural cavity adhesion; and (4) no expected difficulty in femoral vessels cannulation or vena cava occlusion; and (5) a weight above 40 kg.

### Position

After the induction of general anaesthesia, a 35F left-sided double-lumen endotracheal tube was placed to allow for single-lung ventilation. Adult electrodes were attached to the anterior and posterior chest walls as indicated in the instructions (Zoll Medical Corporation, MA, USA). The patients were positioned in the supine position with the right hemithorax elevated to 20°. After systemic heparinization, the femoral vessels were cannulated using a Seldinger technique. The setup of a bypass circuit was initiated by positioning a 16-24F catheter in the abdominal aorta through the right femoral artery. A 28F venous cannula was precisely introduced and positioned into the inferior vena cava (IVC) at the junction with the right atrium (RA) under the surveillance of transoesophageal echocardiography (TEE), and a 16F catheter was percutaneously punctured into right jugular vein (RJV) or inserted into superior vena cava (SVC) through a stab incision in the centre of the purse string suture after pericardiotomy. The arterial and venous cannulas were purchased from Edward Lifesciences corporation (Irvine, California, USA) or Kangxin medical instrument corporation (Changzhou, Jiangsu, China).

### Ports

**Fig. 1:**
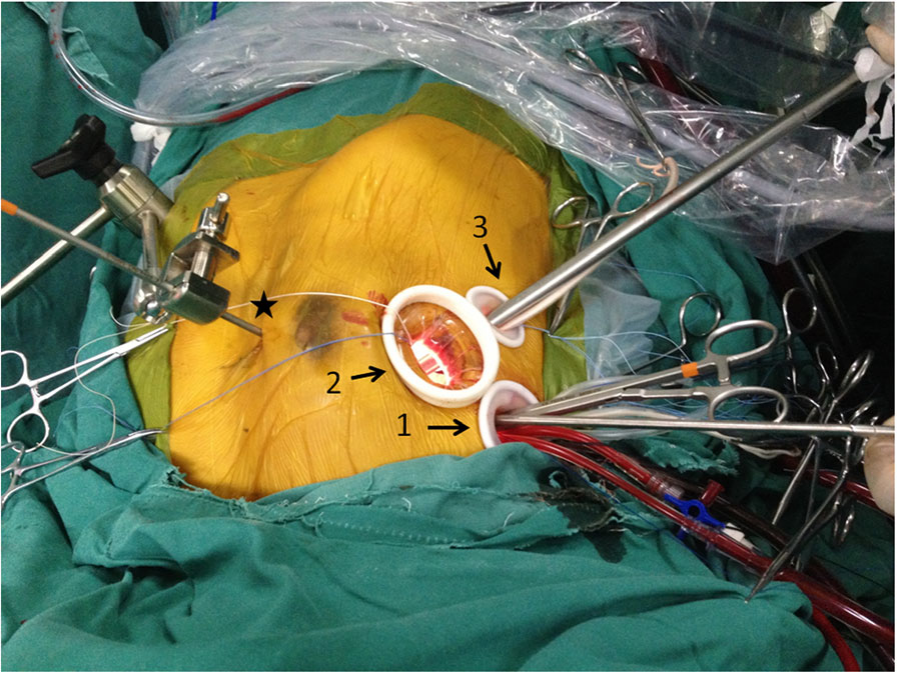
The operative layout for the totally thoracoscopic mitral valve procedure. Drainage of the superior vena cava and right superior pulmonary vein, and cardioplegia perfusion cannula was passed through port 1 directly. Port 2 was located in the fourth intercostal space on a midclavicular line for the entry of instruments, such as scissors and prosthesis, or tissue removal. Port 3 was located in the fifth intercostal space on the right anterior axillary line (arrows). The left atrium was raised by a blade retractor and introduced through a 2-mm shaft penetrating parasternally in the fourth intercostal space and was held on the operating table (asterisk)

Three small incisions and soft tissue retractors were established on the right side of the chest. Port 1 (2–3 cm) was located in the third intercostal space on the right anterior axillary line. This port was the entryway of surgical instruments, such as tissue forceps or suture needles. The drainage for the SVC and right superior pulmonary vein was also passed through the port, in addition to the cardioplegia irrigation tube and aortic clamp forcep. Port 2 (2–4 cm) was located in the fourth intercostal space on a midclavicular line as an entryway for instruments, such as scissors and prosthesis, or tissue removal. Port 3 (2–3 cm) was located in the fifth intercostal space on the right anterior axillary line. This port was for the placement of an thoracoscopy and a subsequent chest drainage tube. A left atrium (LA) blade retractor was introduced into the right chest cavity through a 2-mm shaft penetrating parasternally in the fourth intercostal space and was held on the operating table on the upper end (Fig. 1). A specific thoracoscopy 6800 (Karl Storz Endoskope, Tuttlingen, Germany) was conventionally used.

### Procedures

**Fig. 2:**
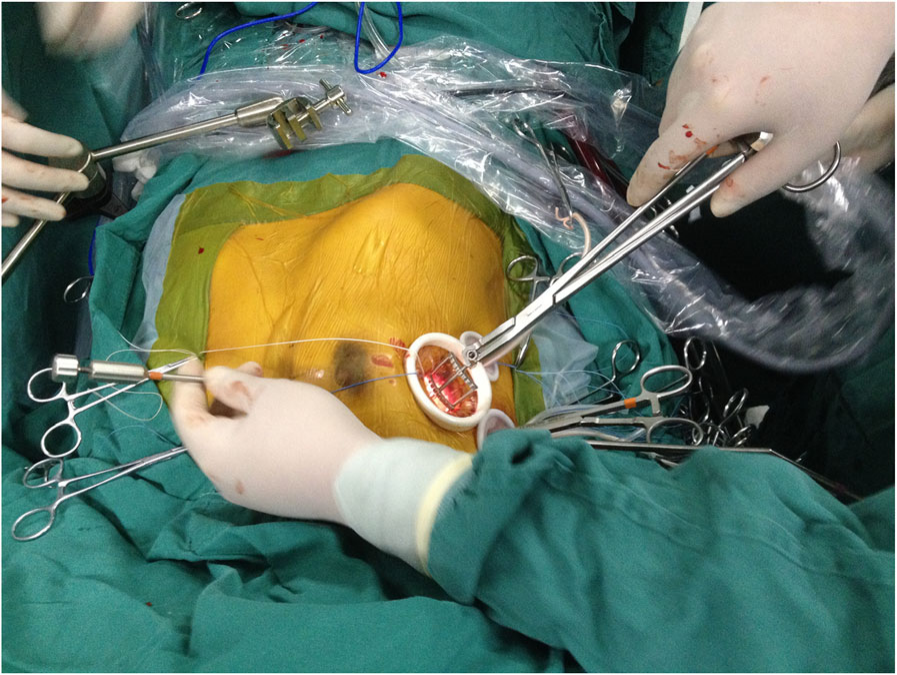
Left atrium exposure by means of a blade retractor. The blade retractor was inserted through port 2 to raise the left atrium with a shaft. The blade retractor was fixed on a 2-mm shaft that penetrated parasternally in the fourth intercostal space and was held on the operating table

**Fig. 3:**
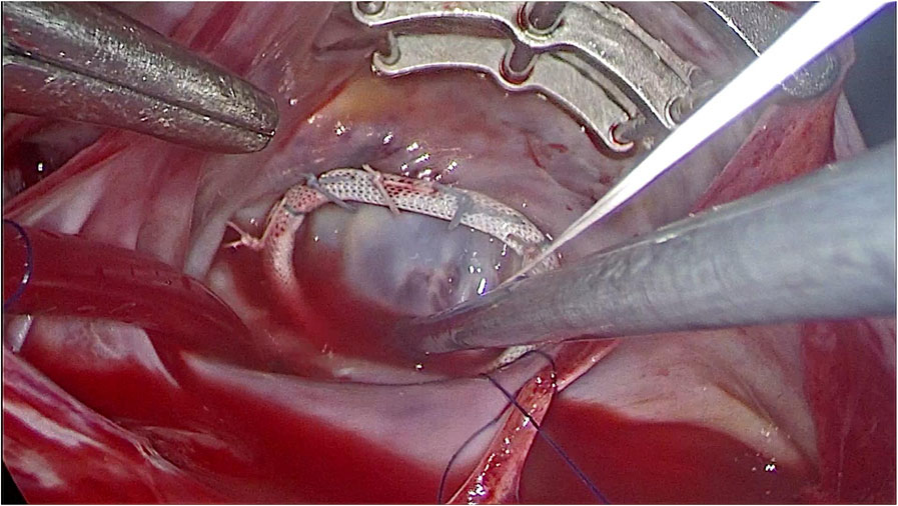
Repair of mitral regurgitation under totally thoracoscopic visual guidance. The annuloplasty ring was implanted for mitral valve repair

Once the previous 3 ports were secured, a pericardiotomy was performed, and 3 to 4 sutures were placed to suspend the pericardium. Caval snares were placed in the SVC and IVC to install total cardiopulmonary bypass (CPB). After CPB initiation and cooling to 32 °C, a transthoracic Chitwood clamp (Scanlan International, MN, USA) was positioned on the ascending aorta. A 7-F long cardioplegia cannula needle (CalMed Technologies, CA) was inserted through port 1 into the aortic root for the delivery of cold blood cardioplegic solution to achieve cardiac arrest [12]. After snaring of the superior and inferior vena cavae, LA traditional access of incision to MV parallel to the interatrial sulcus was opened. The anterior wall of the LA was raised up by means of a specific LA blade retractor as shown in Fig. 2 [13]. The diseased MV was resected and then replaced with a prosthesis valve on the condition of rheumatic mitral stenosis or repaired if mitral regurgitation occurred at the discretion of the operator. Artificial chordae tendineae (Gore-Tex) were used if MV prolapsed or chordae ruptured. The repair was completed by the insertion of an annuloplasty ring as shown in Fig. 3. All annular stitches were exteriorized through the working port and fixed in suture guides. The knots with interrupted sutures were completed extracorporeally and tightened with a knot pusher.

Once the procedure was completed, the LA was closed, leaving the venting suction in place for deairing. If additional procedures on ASD and/or tricuspid regurgitation were performed, the RA was opened. The position and function of the MV prosthesis were confirmed by means of TEE analysis. For patients who suffered from difficulty in heart rebeating or severe arrhythmia after aortic crossclamp release, a pacing wire was introduced into the anterior surface of the right ventricle (RV).

### Perioperative management

Following the operation, patients were monitored in a surgical intensive care unit overnight and were transferred to the wards as soon as they were haemodynamically stable. Additionally, chest X-ray and blood gas analysis were routinely performed to exclude complications in the lungs. Transthoracic echocardiography was performed before discharge, 3 months later and then annually after surgery to assess the postoperational condition.

### Statistical analysis

The short-term outcome consisted of all major adverse events, including intraoperative conversion to sternotomy, re-exploration for bleeding, valve-related reoperation within the same hospital stay, and death. Statistical analysis was performed using SPSS 17.1 software (SPSS Inc., Chicago, IL). Categorical variables were presented as frequencies and percentages, and continuous variables were presented as the mean ± standard deviation.

## Results

### Baseline data and immediate technical feasibility

A total of 53 patients were included in this study. Two patients were excluded by severe indivisible plural adhesion and conversed to median sternotomy. The aetiologies of the mitral lesions and types of surgical procedures are shown in Table 1. The lesions of MV regurgitation happened on the anterior valve in 8 cases and on the posterior valve in 9 cases. Four cases were secondary to ASD, and two cases were concomitant with LA myxoma. There were 38 female patients, almost all of whom were small in stature. All patients underwent 16F cannulation on the SVC and 28F cannulation on the femoral vein. Cannula sizes ranged from 16 to 24F for the femoral artery. Only one patient received a 16F catheter on bilateral femoral arteries due to inadequate bypass flow from the right common femoral artery.

**Table 1:** Baseline data

Characteristics	Totally video-assisted thoracoscopic MV procedure (*n* = 51)
Sex (female)	38
Age (years)	49 ± 14 [41, 60]
Height (cm)	159 ± 8 [154, 163]
Weight (kg)	59 ± 11 [50, 67]
BMI (kg/m^2^)	23.3 ± 3.3 [21.1, 25.9]
Left ventricular ejection fraction (%)	58.9 ± 7.2 [53, 65]
New York Heart Association functional class	2.7 ± 0.5
Coronary artery stenosis (< 50%)	7
Atrial fibrillation	25
Cerebral infarction history	2
Aetiology	51
Rheumatic valve disorder	27
Mitral regurgitation	17
Atrial septum defect (comorbidity)	4 (3)
Infective endocarditis	1
Myxoma	2

Values are given as median (interquartile range)

### Operational data

Mitral valvuloplasty was the priority for all patients with MV regurgitation. Prosthetic rings were conventionally used for all patients if repair was possible. Two patients of these patients underwent mitral valve replacement (MVR) after attempted repair (repair success rate, 88%). Another 34 patients underwent scheduled MVR mainly for rheumatic lesions, only 4 patients received replacement of a biological prosthesis valve, and another 3 patients received concomitant ASD repair. Only 3 patients underwent the Maze procedure with a monopole radiofrequency device. A total of 28 patients (55%) suffered from tricuspid regurgitation and received tricuspid valve annuloplasty (TVP), which was corrected by insertion of an annuloplasty ring.

As shown in Table 2, total operation time, CPB time and ACC time were 251 ± 60 min, 144 ± 39 min and 80 ± 22 min, respectively. Two patients underwent prosthesis valve replacement due to repair failure, and one of these patients underwent a second ACC. One patient experienced serious postoperative perivalvular leakage of a 2.5-cm peri-prosthetic jet under the TEE view, and this patient then completed a corrective procedure under a second ACC. A spontaneous heart beat resumed in 43 patients after the aortic clamp was released (84%). One patient experienced such troublesome haemostasis after sewing the temporary pacemaker wire that a second CPB was resumed, which led to the addition of a right minithoracotomy opposite of the bleeding site on the right ventricle. All of the operations were completed only after no abnormal blood flow kinetics were observed by TEE.

**Table 2:** Operative details

Variable	Totally video-assisted thoracoscopic MV procedure (*n* = 51)
Conversion to minithoracotomy	1
Resumption procedure	2
MVP	17
Gore-Tex artificial chordae tendineae	9
MV Annuloplasty	17
MVR	34
Mechanical	30
Biological Concomitant procedure	4
TVP	28
ASD repair	7
Maze	3
Thrombus removal	3
Warden procedure	1
Operation time (minutes)	249 ± 60 [210, 275]
CPB (minutes)	144 ± 39 [122, 162]
ACC (minutes)	80 ± 22 [66, 86]
Cardiac defibrillation (cases)	8

### Postoperative events

There was only one operative death occurrence. The patient suffered from severe thrombosis on the MV mechanical prosthesis and TV annuloplasty ring and received a second MVR at 7 days after the operation. A low level of platelets while only receiving a half-dosage of low molecular weight heparin without warfarin anticoagulation could have been the cause of death. Transient third-degree atrioventricular conduction block was observed in three (5.8%) patients, who required epicardial temporary pacing. Four patients (8%) experienced postoperative atelectasis, and they recovered uneventfully after respiratory physiotherapy under the guidance of a specialized physical therapist. The New York cardiac function classification class was enhanced at discharge relative to its value before the operation at 2.7 ± 0.5 versus 1.9 ± 0.6, respectively (Table 3, *P* < 0.000).

**Table 3:** Postoperative results

Variable	Totally video-assisted thoracoscopic MV procedure (*n* = 51)
In-hospital mortality	1
Cerebrovascular complication	0
Atelectasis	4
Prolonged intubation (> 48 h)	2
Postoperation IABP implantation	0
Wound infection	0
Lung infection	3
Chest tube drainage (ml, 48 h)*	346 ± 316 [140, 445]
Mechanical ventilation time (hours)*	11 ± 11 [7, 12]
ICU stay (hours)*	23 ± 2 [15, 21]
New York Heart Association functional class at discharge	1.9 ± 0.6
Ejection fraction (%)*	58.5 ± 5.9 [55, 63]

*Values are given as number (interquartile range)

## Discussion

The totally video-assisted thoracoscopic procedure for MV disease is technically challenging, and its application is currently restricted to a handful of experienced operators because it entails the surgeon overcoming a lengthy learning curve [14]. In our department, the technique was performed on 51 cases by one experienced surgeon since the end of 2014. The result was technically feasible, required no transition to median sternotomy, and had a low rate of adverse events.

The totally video-assisted thoracoscopic procedure has been reported as a safe and effective method for repairing simple congenital heart defects with a rapid recovery of life quality and lower pain levels compared with both minithoracotomy and median sternotomy [15]. Meanwhile, lower systemic inflammatory reactions and myocardial damage were also observed after ASD repair with the method [16].

Compared with conventional median sternotomy, the sternum-exempt procedure could not only preserve the integrity of the osseous thoracic wall but also save time on the formidable task of haemostasis as in median sternotomy. Compared with minithoracotomy, which requires a 6–8 cm skin incision in the right inframammary groove to create a small anterolateral “working port” [17], the totally video-assisted thoracoscopic MV procedure had an advantage in terms of minimal invasiveness. The totally thoracoscopic MV procedure, which offers fewer surgical incisions and improved cosmetics, was more attractive to patients than other approaches. The sparing of a rigid incision retractor rendered less injury to chest wall integrity. Nonetheless, high technical demands have impeded the popularization of minimally invasive endoscopic MV surgery.

It is too challenging to widely introduce this procedure because it is still difficult to perform for a large majority of surgeons. The survey of surgeons who were experienced in minimally invasive MV surgery showed that 90 % of the respondents believed that more than 20 cases were required to gain familiarity with the procedure [18]. Our department introduced a skilful cardiac surgeon who was well versed in performing the MV procedure with the guidance of a video-assisted thoracoscope instead of port-access minithoracotomy and robotic assistance. He had already carried out nearly 100 cases of MV repair/replacement from 2012 to 2014.

There were some disadvantages and pitfalls in terms of the characteristics of the totally video-assisted thoracoscopic MV procedure. Compared with the three-dimension vision under the totally direct open procedure, the manipulation zone lacked a stereoscopic experience. The field of vision during the operation was narrowed down by enlarged thoracoscope so that the surgeon had increased difficulty during manipulation. Each knotting entailed crossing of the sutures outside of chest cavity and then pulling down the knots with the assistance of a knot pusher.

There were some technical limitations in its clinical application. One of the key elements for procedural success was CPB establishment through femoral artery-vein bypass. Patients had a low body weight with smaller femoral blood vessels, which might hinder the insertion of venous or arterial cannulas for optimal CPB. As Table 1 indicates, the great majority of MV patients in our region were female and rheumatic, short in statue, with a BMI ranging from 18.1 to 30.8. Six patients received a 16F femoral artery cannula, and only one case underwent bilateral femoral artery cannulation due to hypoperfusion. The SVC was completely cannulated with a 16F cannula, which was inserted through the RJV during the initial phase of the programme. Due to the scar effect on the neck and the associated risk of penetrating into the pleural cavity, the plan was terminated after performing the first 11 cases in the middle of 2015. Instead, the operator completed the catheterization of the SVC directly through port 1 under the view of thoracoscopy. The femoral vein was successfully cannulated with a 28F cannula for IVC drainage. This satisfactory perfusion result could be due to the lower morbidity rate of peripheral artery disease in this population. Elective preoperative coronary angiography was often performed on the right radial artery, which avoided possible intima injury to the femoral artery.

The second element was heart arrest and cardiac protection. In our study, a lengthened cardioplegia perfusion cannula and transthoracic aortic clamp were introduced conventionally and smoothly. It was reported that intraluminal aortic clamping was applied by means of a sophisticated device consisting of a three-lumen catheter named an Endoclamp, which enabled simultaneous occlusion of the aorta, antegrade delivery of cardioplegia, and venting through the aortic root [19]. The minimally invasive approach should be excluded in patients with aortic regurgitation (AR) greater than grade I because of the risks of inadequate cardioplegia delivery and left ventricular distension [12]. No evidence in our study demonstrated that retrograde perfusion per se during minimally invasive MV surgery increased the incidence of cerebrovascular accidents [20].

The requirement of rethoracotomy for bleeding represented a major drawback of minimally invasive MV surgery because the entire operative field could not be directly visualized. In this study, there was one case undergoing transition to mini-thoracotomy for bleeding at the site of temporary pacemaker wire implantation, but no reexploration for bleeding happened after chest wall closure. This excellent outcome was attributable to overcoming the learning curve and innovative measure by the operator, who fixed the pacemaker wire with Teflon pressed with a titanium clip at both ends [21]. It was also routine for the operator to use a thoracoscope to inspect the inner thoracic wall thoroughly before closing the incision. It had been recorded that the rate of reexplorations for bleeding fell; it was reduced from 8.2 to 1.9% after 300 operations [11]. In contrast, we observed one case in which that patient died from prosthesis thrombosis because of insufficient anticoagulation. Due to fear of bleeding on the condition of thrombocytopenia and continuous excessive drainage, we did not regularly prescribe warfarin for prosthesis anticoagulation. The subsequent MV prosthesis replacement re-do procedure was ineffective, and the patient died from coagulation complications soon after the procedure. Granted that only two patients suffered reoperation after ACC release, the resultant operative quality of totally video-assisted thoracoscopic MV surgery could be on par with the conventional median sternotomy approach.

Regarding feasibility, chest wall deformity was restricted to perform the totally video-assisted thoracoscopic procedure to some extent. Due to case limitation and careful patient selection, we were not confronted with any cases of serious chest deformity or secondary port-access video-assisted thoracoscopic procedures. Nevertheless, the mere presence of uncorrected congenital chest wall deformities should not deter surgeons from minimally invasive cardiac surgery [22]. Loose pleural adhesion did not restrict the feasibility unless indivisible adhesion existed. We also experienced several cases of painstaking synechiotomy and then completed the MV procedure under the guidance of thoracoscope. It had been shown that the outcomes in redo-port access surgery after previous port access surgery were favourable [23]. In our population, two cases required re-do surgery of a previous MV procedure were successfully completed in patients who underwent percutaneous balloon mitral valvuloplasty and MVR at median sternotomy. To some extent, the procedure from right thoracotomy access should be the optimal choice for re-do MV patients because post-sternum adhesion is avoided. The LA blade retractor used to pull up the LA anterior wall was held by a shaft that penetrated into chest cavity rather than through the main incision [24, 25]. This approach could minimize the size of the main incision, which merely allowed the prosthesis to enter into the port smoothly. Meanwhile, the additional penetrating point for the shaft did not increase the cosmetic burden.

Concomitant TVP was not associated with a risk-adjusted increase in mortality, regardless of TR severity. A more liberal approach to TVP at the time of MVR might be justified when long-term benefits are thought to outweigh incremental short-term morbidity risk [26]. In our study, we completed MV procedures associated with TVP in 28 cases. The operator also performed the Warden procedure in one case who suffered from ASD combined with supracardiac partial anomalous right upper pulmonary venous connection to SVC. A giant self-pericardium patch was added to the RA as an atrial septum to separate the total pulmonary venous connection into the LA [27].

## Conclusions

We completed 51 cases of MV procedures totally by thoracoscopic visual guidance. The aetiology of these MV diseases was comprised of mitral regurgitation, rheumatic mitral disorders, congenital heart defect or left atrial myxoma. Through three incisions that were 2–4 cm length and one penetrating point in the right chest wall, mitral valve replacement or repair was successfully administered by an experienced surgeon. The additional procedures included LA thrombus removal, Cox-maze radiofrequency, ASD repair, and TV annuloplasty. Apart from its minimal invasiveness characteristics and wide range of clinical applications, the MV procedure under the assistance of thoracoscopic visual guidance and TEE surveillance was also a safe and effective operation approach. In total, the thoracoscopic procedure for conventional MV diseases is worth being advocated broadly, and more experienced surgeons should be trained on this procedure due to its high-demand in terms of manipulation skills and three-dimensional conception. In summary, the totally thoracoscopic procedure for MV disease by an experienced operator is feasible, safe, effective, and merits widespread adoption.

## Abbreviations

ACC: aortic cross clamping
AR: aortic regurgitation
ASD: atrial septal defect
CPB: cardiopulmonary bypass
IVC: inferior vena cava
LA: left atrium
MV: mitral valve
MVR: mitral valve replacement
RA: right atrium
RJV: right jugular vein
RV: right ventricle
SVC: superior vena cava
TV: tricuspid valve
TVP: tricuspid valve annuloplasty
